# Common genetic variants in *HIVEP3* are associated with cognitive SuperAging in the Amish

**DOI:** 10.1002/alz70855_104917

**Published:** 2025-12-24

**Authors:** Daniel A. Dorfsman, William K. Scott, Michael B. Prough, Alex V. Gulyayev, Laura J. Caywood, Jason E. Clouse, Sharlene D. Herington, Susan H. Slifer, Larry D Adams, Renee A. Laux, Yeunjoo E. Song, Audrey Lynn, Sarada L. Fuzzell, Sherri D. Hochstetler, Kristy L. Miskimen, Leighanne R. Main, Ping Wang, Yining Liu, Noel C. Moore, Paula K. Ogrocki, Alan J. Lerner, Jeffery M Vance, Michael L Cuccaro, Jonathan L Haines, Margaret Pericak‐Vance

**Affiliations:** ^1^ John P. Hussman Institute for Human Genomics, University of Miami Miller School of Medicine, Miami, FL, USA; ^2^ Dr. John T. Macdonald Foundation Department of Human Genetics, University of Miami Miller School of Medicine, Miami, FL, USA; ^3^ Department of Population and Quantitative Health Sciences, Case Western Reserve University School of Medicine, Cleveland, OH, USA; ^4^ Department of Population and Quantitative Health Sciences, Institute for Computational Biology, Case Western Reserve University, Cleveland, OH, USA; ^5^ Department of Genetics and Genome Sciences, Case Western Reserve University School of Medicine, Cleveland, OH, USA; ^6^ Cleveland Institute for Computational Biology, Case Western Reserve University, Cleveland, OH, USA; ^7^ Department of Neurology, University Hospitals Cleveland Medical Center, Cleveland, OH, USA; ^8^ Department of Neurology, Case Western Reserve University School of Medicine, Cleveland, OH, USA

## Abstract

**Background:**

Studies of older adults with exceptional cognitive performance can enhance understanding of the mechanisms that protect against Alzheimer's disease (AD). Cognitive SuperAgers (SA) are individuals aged 80 and above with above‐average episodic memory performance exceeding norms for middle‐aged adults. This study integrates family‐ and association‐based analytical approaches to identify genetic variants associated with SA in the Midwestern Amish population.

**Methods:**

A comprehensive neuropsychological evaluation was conducted among adult Amish participants (*N* = 515). SA were defined as those aged ≥ 80 with episodic memory task performance at or above the mean for ages 35‐44, and non‐episodic memory tasks within one standard deviation of the mean or better for the participant's age (*N* = 83). SA were grouped into 16 pedigrees for parametric and non‐parametric linkage analysis allowing for locus heterogeneity in MERLIN. Variants located in regions exhibiting HLOD or LOD* scores ≥ 3 were tested for association with SA in GENESIS. Comparison groups included cognitively unimpaired, age‐matched non‐SA individuals (CU 80+, *n* = 157) and individuals with AD (*n* = 40). Significance thresholds for each region were determined using SimpleM, which estimates the number of independent tests in the presence of high linkage disequilibrium.

**Results:**

Linkage analysis identified HLOD scores > 3 on chromosomes 1 (HLOD = 3.10, GRCh38 44.6 Mb), 2 (HLOD = 3.92, 202.9 Mb), 7 (HLOD = 3.14, 30.2 Mb), 16 (HLOD = 3.18, 22.7 Mb), and 20 (HLOD = 3.71 16.7). Regional analysis revealed significant associations for eight correlated variants in the *HIVEP3* gene on chromosome 1, comparing SA to AD (peak signal at rs12734651, OR = 0.24, *p* = 6.46 x 10^‐6^). These variants were nominally associated when comparing SA to CU 80+ (rs12734651, OR = 0.61, *p* = 0.032).

**Conclusions:**

This study identified variants in *HIVEP3* associated with SA. Previous studies have linked variants in *HIVEP3* to increased risk of AD, as well as with hippocampal volume and cognitive trajectories in unimpaired adults, suggesting that variants in *HIVEP3* may influence both AD risk and cognitive performance in unimpaired individuals. These findings indicate that *HIVEP3* represents a plausible candidate for further investigation into the mechanisms promoting exceptional cognitive performance in older adults.

